# Reinforcement of suspensory ligaments under local anesthesia cures pelvic organ prolapse: 12-month results

**DOI:** 10.1007/s00192-013-2281-x

**Published:** 2013-12-07

**Authors:** Yuki Sekiguchi, Manami Kinjo, Yoshiko Maeda, Yoshinobu Kubota

**Affiliations:** 1 Yokohama Motomachi Women’s Clinic LUNA, 3-115 Hyakudan-kan 5F, Motomach, Nakaku, Yokohama, 231-0861 Japan; 2 Department of Urology, Yokohama City University Graduate School of Medicine, Yokohama, Japan

**Keywords:** TFS, POP, Pelvic organ prolapse, Day surgery, Adjustable minisling, Local anesthetic surgery, Day surgery

## Abstract

**Introduction and hypothesis:**

In 2005, a new minimally invasive procedure, the tissue fixation system (TFS) was reported. Like TVT (tension-free vaginal tape), the TFS works by creating a foreign body collagenous tissue reaction that reinforces a weakened pelvic ligament. The objective was to assess the effectiveness and perioperative safety of TFS in a day surgery clinic for the treatment of pelvic organ prolapse (POP).

**Methods:**

The TFS tape was applied in a tunnel adjacent to natural ligaments to repair the anterior cervical ring and cardinal ligament, paravaginal tissues and uterosacral ligaments under local anesthesia/sedation.

We prospectively studied 60 patients, mean age 67, between October 2008 and February 2010 at Women’s Clinic LUNA. Levels of POP were grade 2 (*n* = 20; 7 %), grade 3 (*n* = 30; 55 %), and grade 4 (*n* = 4; 7 %) according to the ICS POPQ classification. Fifty-four patients (90 %) who underwent a total of 162 POP operations presented for review. Follow-up was performed at 12 months. We defined surgical failure according to the ICS POPQ classification. We used prolapse quality of life (P-QOL) questions for QOL measurement.

**Results:**

Ninety-eight percent of patients were discharged on the day of surgery. Of the162 TFS operations reviewed, 157 were successful and 5 failed. The 5 failed operations comprised 4 cystoceles and 1 rectocele. Two patients developed cervical protrusions at the introitus at 6 months with no prolapse of the uterine body. We found 5 cases of erosion in 162 tape insertions. The total number of patients who had no complications, no surgical failures, no erosions, no sensation of bulging, and no cervical protrusions was 47 (87 %).

**Conclusions:**

The TFS uses the same surgical principle for repair as the TVT; this principle vastly minimizes the volume of mesh used, erosions, and other complications.

## Introduction

**Fig. 1:**
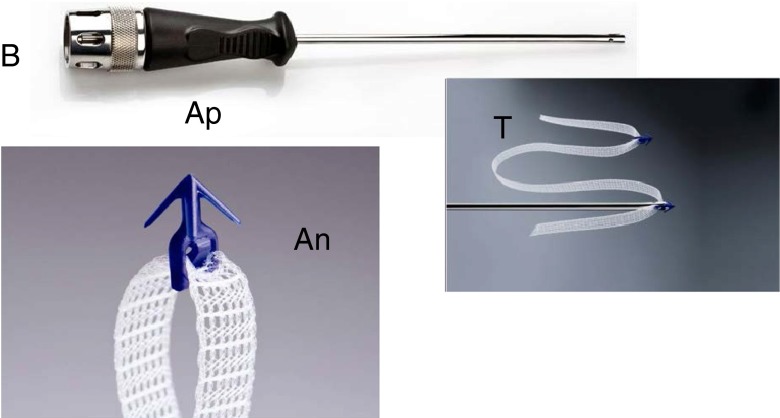
The tissue fixation system (TFS). The TFS consists of two anchors (*An*) joined by a polypropylene mesh tape (*T*) passing through a one-way trapdoor at each base, which allows tightening. The anchor is fitted on top of the TFS applicator (*Ap*). The anchor is released into the tissues by activating the TFS applicator button (*B*)

Japan has an ageing population. As a consequence, an increasing number of patients are developing pelvic floor laxity. Because of the presence of many collateral health problems, all of which vastly increase the risks of surgery, minimally invasive operations are preferable for safety and quality of life. Another concern is conservation of the uterus. Hysterectomy is widely performed concomitantly whenever the uterus is significantly prolapsed. However, hysterectomy involves major surgery with sometimes major complications. Furthermore, it is suggested that hysterectomy itself may be a cause of pelvic organ laxity, as the descending uterine artery is a major blood supply to the uterosacral and cardinal ligaments. There is no clear evidence supporting the role of hysterectomy in improving surgical outcome [1]. In 2005, a new minimally invasive methodology that permits uterine conservation, the “tissue fixation system (TFS)” was reported (Fig. 1) [2]. A 7-mm wide, non-stretch monofilament TFS tape is applied entirely via a single vaginal incision, thus avoiding perforation of suprapubic, or perineal skin. Like TVT (tension-free vaginal tape), the TFS works by creating a foreign body collagenous tissue reaction that reinforces a weakened pelvic ligament [3]. It has been successfully applied to reinforce pelvic ligaments and fascias for cystocele, vault prolapse, and perineal body repair [2, 4, 5] and stress incontinence [6, 7], according to an anatomical classification [8]. This procedure can be carried out on a day surgery basis. The aim of this study was to assess the effectiveness and perioperative safety of the tissue fixation system (TFS) tensioned minisling used as a minimally invasive surgical repair in a day surgery clinic for the treatment of pelvic organ prolapse (POP).

## Materials and methods

We studied prospectively 60 patients who had undergone 180 site-specific TFS POP operations on a day surgery basis between October 2008 and February 2010 at Yokohama Motomachi Women’s Clinic LUNA. This study was approved by the Ethics Committees of the Yokohama Motomachi Women’s Clinic LUNA in 2006. Written informed consent was obtained from all patients.

The prolapse was staged according to the ICS POPQ classification [9, 10] as follows: 0—no prolapse is demonstrated during maximal straining; I—the most distal portion (leading surface) of the prolapse is > 1 cm above the level of the hymen (< −1 cm); II—the most distal portion (leading edge) of the prolapse is ≤ 1 cm proximal to or extends 1 cm through the plane of the hymen (≥ −1 cm, but ≤ + 1 cm); III—the most distal portion of the prolapse is > 1 cm below the hymen, but no further than 2 cm less than the TVL (there is not complete vaginal eversion; > +1 cm, but < + [TVL −2] cm); IV—complete eversion of the vagina (≥ + [TVL −2] cm).

We evaluated general quality of life (QOL) affected by POP according to the Prolapse Quality of Life (P-QOL) questionnaire (Q)2, at which point 1 is “not affected at all,” point 2 is “affected slightly,” point 3 is “affected considerably,” and point 4 is “affected very much.”

**Fig. 2:**
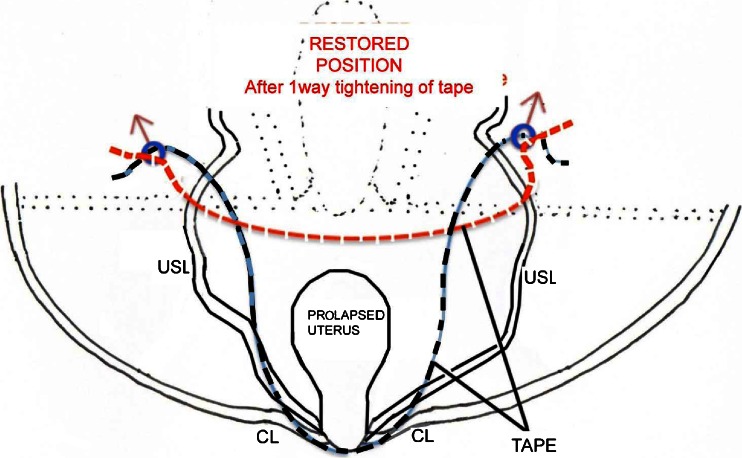
Repair principle using tensioned tapes: schematic diagram. Example: how the TFS repairs the uterosacral ligaments (*USL*). *Blue solid line*: tape prior to tightening; *red broken lines*: tape after tightening; *black broken lines*: replaced organ, ligaments, and connective tissues. *CL* lax cardinal ligament

The TFS (TFS Surgical, Adelaide, Australia) is an adjustable sling device for stress urinary incontinence and pelvic organ prolapse (Fig. 1). It consists of two polypropylene 4 pronged plastic anchors attached to a non-stretch lightweight monofilament polypropylene mesh tape. The anchors have a pull-out strength of approximately 2.5 to 3 kg each. Their mode of action is like a grappling hook. At the base is a one-way tensioning system that uniquely shortens and reinforces laterally displaced ligaments and fascia to the correct anatomical position (Fig. 2). Shortening an overstretched ligament allows proper function of any muscle that contracts against such a ligament, Fig. 2. Histology studies in rats demonstrated that the anchor is entirely covered with collagenous tissue within 2 weeks of implantation. This prevents any movement and exposure of the anchor [4].

### Local anesthetic technique

Patients were given hydroxyzine hydrochloride 25 mg and atropine sulfate 0.5 mg i.m., and diclofenac sodium 50 mg p.r. before surgery. The operations were performed under local anesthesia (LA) by two surgeons, using 1 % xylocaine 40 ml + physiological saline 160 ml + vasopressin 40 units. Patients were additionally given midazolam 5 mg intravenously.

### Surgery

**Fig. 3:**
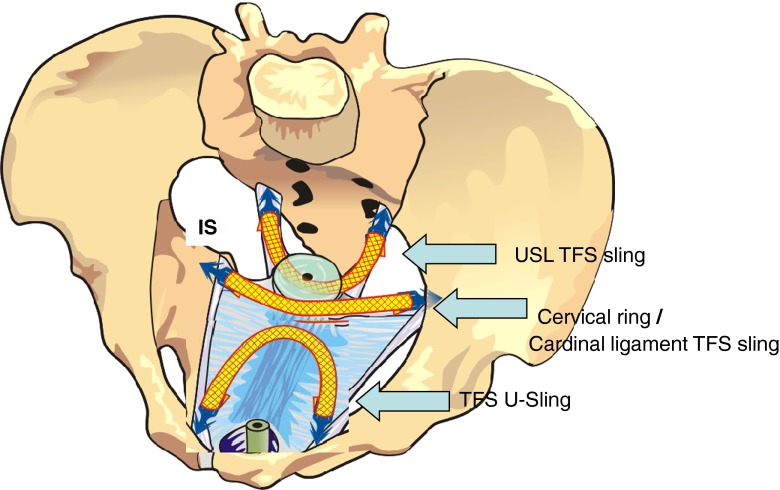
TFS for pelvic organ prolapse (POP). *Cervical ring/cardinal ligament sling* the anchors were put along the cardinal ligament beyond the lateral sulcus. *TFS U-Sling* The anchors were placed below the pubic ramus, extending onto the medial aspect of the obturator fossa, in the position of the ATFP insertion. *USL TFS Sling* The anchors were placed approximately 2 cm distal from the insertion of the uterosacral ligaments (USL) into the sacrum

All patients underwent implantation of 3 TFS tapes for repair of the cardinal ligament, the arcus tendineus fasciae pelvis (ATFP), and the uterosacral ligaments (Fig. 3).

First, we performed a cardinal ligament TFS sling for restoration of the cardinal ligament/cervical ring defect (also known as the “transverse defect”) (Fig. 3) [8]. The surgical principle underpinning this operation is similar to that of a Manchester repair, except that the TFS also re-attaches the cervix to the pelvic side wall (Fig. 3). The one-way tensioning system of the anchors shortened and re-attached the elongated and laterally displaced cardinal ligament to the anterior part of the cervix and pelvic side wall. The tissue reaction generated by the tape “reglued” the pubocervical fascia to the anterior cervical ring. A 2.5- to 3-cm horizontal incision was made in the vesical fold 1 cm above the cervix. The bladder was dissected clear of the vagina and cervix. Using dissecting scissors with the tips everted and firmly applied to the vagina, a channel was made along the cardinal ligament just beyond the lateral sulcus. The dissection plane was about 2 cm above the ischial spine. The TFS applicator was inserted into the tunnel and the anchor released [5]. After waiting 10 s to allow rebound elastic closure of the tunnel, the tape was tugged laterally to set the anchor. The procedure was repeated on the contralateral side. The tape was tightened until a resistance was felt, indicating return of muscle contractility.

We then performed the U-sling operation for paravaginal repair (Fig. 3) [5]. The surgical principle underpinning this operation is to reinforce the ATFP (arcus tendineus fasciae pelvis), re-attach the pubocervical fascia to the ATFP (correction of the lateral defect), and to provide a transverse neofascial “beam” to reinforce the damaged central pubocervical fascial defect [5]. Keeping the scissors pressed against the vagina, again under tension, a channel was made below the pubic ramus, extending onto the medial aspect of the obturator fossa, in the position of the ATFP insertion. The applicator and anchor/tape were inserted and tightened as described previously.

Finally, the uterosacral ligament (USL) TFS sling operation was performed (Fig. 3). The surgical principles underpinning this operation are to use the one-way tensioning system of the anchors to shorten the laterally displaced uterosacral ligaments, lift the uterus back into its original anatomical position (Fig. 2), and using the tissue reaction generated by the tape, to “reglue” the rectovaginal fascia to the posterior cervical ring. A 2.5-cm transverse incision was made 2 cm below the cervix. The uterosacral ligaments (USLs) or their remnants were identified and grasped with Allis forceps. The USLs were confirmed by rectal examination. Then, under tension, scissors angled at 30° perforated the uterosacral ligaments approximately 2 cm distal to their insertion points to the sacral bone in a tunnel sufficiently wide to accommodate the TFS applicator. The applicator and anchor/tape were inserted and tightened as described previously.

Blood loss was measured in milliliters from the suction bottle and preweighed swabs.

### Outcome measures

The primary outcome measure was re-assessment of the patient by vaginal examination using the POPQ system at 3, 6, and 12 months. We defined surgical failure where points Ba, Bp or C, the distal portion (leading surface) of the prolapse were > −1 cm above the level of the hymen during straining. Secondary outcome measures were symptom change, blood loss, and report of complications. The results as reported concern only the 12-month review, which included the questionnaires.

### Data analysis and statistics

Patient characteristics and symptoms were summarized using descriptive statistics for continuous variables presented with means and standard deviations as appropriate. Categorical data were presented as rates and percentages. Anatomical outcome and recurrence rates were assessed by a POP-Q examination considered to be the primary outcome. Surgical failure (relapse) was defined as the presence of prolapse stage 2 or more in the middle compartment with prolapse complaints and/or redo surgery at 1-year follow-up according to the POPQ classification. We used a *t* test from software PASW statistics 18 (SPSS Inc., Japan) to assess statistical significance at the two-sided 5 % level (*p* < 0.05).

## Results

Sixty POP patients underwent corrective surgery between October 2008 and February 2010. Six patients were not contactable, either by letter or by repeated telephone calls. Fifty-four patients (a response rate of 90 %) agreed to attend for 12-month review. These patients were assessed by POP-Q examination and questionnaires. The average patient age was 67 years (range 44 to 87 years). Mean BMI was 24.1 (±3.7) and parity 2.4 (0–4). Levels of POP were stage 2 (*n* = 20; 37 %), stage 3 (*n* = 30; 55 %), and stage 4 (*n* = 4; 7 %). Average operative time was 86 min (minimum 40 min, maximum 135 min) and average blood loss 21 g. (minimum 4 g, maximum 136 g). Fifty-three patients (98 %) were discharged on the day of surgery. One patient with a hematoma was transferred to a neighboring hospital for observation purposes only. There was no significant morbidity and no transfusion of blood was required. One patient required intermittent self-catheterization for urinary retention. She was able to void normally after 2 weeks.

We defined surgical failure according to the ICS POPQ classification. Fifty-four patients underwent a total of 162 separate TFS operations. Of these, 157 were successful and 5 failed. The total number of patients who had no complications, no symptoms of bulging, no surgical failures, no erosions, and no cervical protrusions was 47 (87 %). The 5 failed operations comprised 4 cystoceles and 1 rectocele. In addition, 2 patients developed cervical protrusions at the introitus 6 months after TFS surgery. There was no prolapse of the uterine body; thus, only the elongated cervix was amputated. We found 2 cases of tape erosion and 3 tape/anchor extrusions associated with prolapse relapse in a total of 162 operations. We believe that 3 of the surgical failures were caused by inadequate application of the anchor, resulting in slippage and erosion of the tape. There were no cases of primary anchor migration. Two of the erosions were not associated with surgical failures. Four of the 5 failed cases underwent re-operation and were cured by TFS POP operations. Three patients developed de novo stress incontinence. These were cured by a subsequent mid-urethral TFS sling operation.

The improvements in urinary-related QOL are summarized in Table 1. There were statistically significant improvements in pollakisuria (urinary frequency), urgency, urge urinary incontinence, and stress urinary incontinence. The average score of P-QOL Q2“QOL affected by POP” changed between pre- and post-operation from 3.77 ± 0.78 to 2.44 ± 0.61 (*p* < 0.01).

**Table 1:** Improvement in urinary-related QOL

Number of P-QOL	Symptom	Average points ± SD (pre-operation)	Average points ± SD (post-operation after 1 year)	*p* value
Q3	Pollakisuria (urinary frequency)	3.34 ± 1.04	2.74 ± 0.79	< 0.01
Q4	Urgency	3.05 ± 0.99	2.66 ± 0.67	< 0.05
Q5	Urge urinary incontinence	2.89 ± 0.83	2.62 ± 0.67	< 0.05
Q6	Stress urinary incontinence	3.22 ± 0.97	2.82 ± 0.79	< 0.05

## Discussion

This is the first report of TFS POP repair under local anesthesia (LA) with same-day patient discharge. The local anesthetic surgical technique we used was made possible by the minimally invasive nature of the TFS system and its anatomical accuracy. Importantly for an LA technique, the TFS allows uterine conservation. Our experience is that the TFS adjustable minisling operations are safe and effective. They are ideally suited to older women as there Is minimal disruption of activities and because the surgical methodology minimizes serious post-operative complications such as deep vein thrombosis, pulmonary embolism, and complications from the intercurrent diseases associated with old age. Of course an arrangement with neighboring hospitals is required for patient transfer in the case of an emergency.

The primary outcome result on a patient operated basis (no complications, no surgical failures, no symptoms of bulging, no erosions, no cervical protrusions) was 87 %. This compares favorably with those of a large RCT for cystocele reported by Altman et al. [11] who reported the primary outcome results (POPQ staging 0–1 plus no symptoms of prolapse ) of 60.8 % in women treated with transvaginal mesh repair (*n* = 200) compared with 34.5 % in women treated with native tissue colporrhaphy (*n* = 189). Mean operating time for our patients was 86 min for three site-specific TFS operations compared with 56 min for the cystocele mesh repair [11]; our patients lost slightly less blood and there were no bladder perforations. The eroded TFS tapes did not require surgical re-intervention: rather, the eroded part was trimmed, like an eroded TVT tape.

We attribute our high cure rate to the novel surgical methodology of the TFS, which requires accurate diagnosis and precise anatomical restoration of damaged ligaments. The cardinal and uterosacral ligaments are the principal ligamentous supports of the uterus and these insert into the cervical ring [12]. Also, attached to the cervical ring is the pubocervical fascia (PCF) anteriorly and the rectovaginal fascia (“RVF” or “Denonvilliers’ fascia”) posteriorly. The PCF inserts distally into the ATFP. The RVF inserts distally into the perineal body, and proximally into the cervical ring and levator plate muscles [13].

Normal fascial attachments between organs and the suspensory ligaments allow the pelvic muscles to stretch the upper part of the vagina and the uterus downward and backward for normal support and function of the organs [8]. Childbirth, age, and congenital collagen defects are major causes of the breakdown of this mechanism. The baby’s head descending down the vagina stretches the connective tissue supporting structures (ligaments and fascia) laterally, thereby causing laxity. Lateral displacement of ligaments and associated connective tissues may cause the bladder, uterus, and rectum to herniate through the levator hiatus as a cystocele, uterine prolapse, rectocele, with or without urinary and bowel symptoms [8, 12]. Our data show an improvement in bladder function as well as prolapse cure.

We attribute symptom improvement to the tensioning properties of the TFS, which restores the laterally displaced pelvic ligaments and fascias to their former condition, thereby restoring not only organ support, but function [6]; also, because the TFS tape traverses the levator hiatus transversely, as well as creating artificial collagenous neoligaments, we hypothesize that it “re-glues” the organs to the levator hiatal muscles, as demonstrated previously in experimental animals [14]. This would limit the lateral “ballooning” of the hiatal muscles observed during straining [15].

The importance of collagenous attachments of the organs to levator hiatus was first described by Robert Zacharin in 1963 [16].

In 1997, the infracoccygeal sacropexy, also known as the posterior-IVS (PIVS), a minimally invasive procedure for the restoration of uterine or apical prolapse, was introduced [17]. This procedure was safer and simpler than previous procedures for apical prolapse. Importantly, it preserved the uterus. However, PIVS operation was not entirely anatomical, as it attached the apex to the levator muscles/sacrospinous ligament (SSL) rather than to the uterosacral ligaments, which were sited some 3 cm cranial to the SSLs. Nor did the PIVS address anterior vaginal wall defects, leading to anterior wall prolapse (cystocele) in 16 % of patients [17], and it did not re-attach the organs to the levator hiatus. We believe that one factor in the TFS attaining a higher cure rate than large mesh is the potential ability of the TFS to re-attach the organs to the hiatus.

All TFS sling operations are a direct evolution of the TVT procedure, in that they work by using only a short thin tape applied through a tunnel. By using the TFS, we were able to reinforce the cardinal ligament, pubocervical fascia, ATFP and uterosacral ligaments, all major supports of the uterus and the anterior and posterior vaginal walls. The TFS sling’s unique ability to tension, shorten, and reinforce weakened elongated ligaments (Fig. 2), was an important factor in restoring the tension needed for the muscle forces to be able to restore function, as no muscle can contract efficiently against a loose connective tissue insertion [18].

A cystocele may reoccur, or occur de novo following repair of uterine prolapse, even when a cystocele repair is performed at the same time by anterior colporrhaphy [11, 17]. Because of this, many surgeons prefer to insert large meshes into the organ spaces to prevent organ descent. This “glues” the vagina to the bladder or rectum, causing scarring and contraction, and preventing distension during intercourse, all major causes of the dyspareunia reported with large mesh insertion [11, 19]. Large volumes of mesh lead to more scarring, erosions, infections, hematomas, even organ perforation and fistulas, ultimately resulting in the recent FDA warnings of major complications with such meshes [20].

The TFS method uses a different bioengineering approach to that of large mesh implants: reinforcing existing suspensory ligaments with a precisely inserted tape via a tunnel adjacent to the ligament. The scar tissue created by tissue reaction to the implanted tape reinforces the damaged ligament [14]. Scar tissue has a breaking strain of 1,300g/cm^2^ [21]. This explains how we were able to cure even 4th degree POPs using small thin lengths of tape.

Tensioning the tape provides strength in the manner of a wire suspension bridge; spanning with tapes (Fig. 3) reinforces the anterior vaginal wall like joists of a ceiling reinforcing a plasterboard. Unlike large sheets of mesh, the organ spaces are preserved and adhesions are avoided. Thus, antero-posterior elasticity is largely maintained, allowing the antero-posterior movements so important for efficient vesico-urethral and anorectal closure (continence) and evacuation [8]. Adhesions are also potent causes of dyspareunia, as they prevent the vagina from stretching independently of the rectum.

We believe that failure in 5 cases was due to a loose tape caused by incorrect placement of the anchor. This may have been because of learning curve issues, or weak pelvic connective tissues. Because the subsequent TFS surgery was successful, we attribute the failure to the former, surgical error.

Our erosion rate, 5 per 162 tapes implanted, is consistent with published erosion rates for the TVT [22]. It is also consistent with the differentiation between mid-urethral tapes for urinary stress incontinence (acceptable) and large mesh for POP (questionable) according to the recent 2011 warning by the FDA [20].

## Conclusions

The TFS applies the same surgical principle for repair as the TVT, whereby a thin polypropylene tape is placed in a tunnel to reinforce a damaged (pubo-urethral) ligament. We applied the same principle to repair the cardinal and uterosacral ligaments. Unlike native tissue repairs, which remove and suture centrally bulging defects, the TFS conserves vaginal tissue, attaches the organ to the pelvic side wall and to the hiatus. Its one-way system shortens the elongated ligaments to elevate the organ. Because the tape inserted is of such a small volume, the risk of erosion, contraction, dyspareunia, and system malfunction are likely to be decreased. The results are encouraging, but longer follow-up data are required.

## References

[1] Neuman M, Lavy Y (2007). Conservation of the prolapsed uterus is a valid option: medium term results of a prospective comparative study with the posterior intra-vaginal slingoplasty operation. Int Urogynecol J Pelvic Floor Dysfunct.

[2] Petros PEP, Richardson PA (2005). The tissue fixation system posterior sling for repair of uterine/vault prolapse-a preliminary report. Aust N Z J Obstet Gynaecol.

[3] Papadimitriou JM, Petros PEP (2006). Biocompatible properties of surgical mesh using an animal model. Aust N Z J Obstet Gynaecol.

[4] Petros PE, Richardson PA (2008). Midurethral tissue fixation system (TFS) sling for cure of stress incontinence–3 year results. Int Urogynecol J Pelvic Floor Dysfunct.

[5] Petros PEP, Richardson PA, Goeschen K, Abendstein B (2006). The tissue fixation system (TFS) provides a new structural method for cystocoele repair—a preliminary report. Aust N Z J Obstet Gynaecol.

[6] Sekiguchi Y, Kinjyo M, Inoue H, Sakata H, Kubota Y (2009). Outpatient mid urethral tissue fixation system sling for urodynamic stress urinary incontinence. J Urol.

[7] Sivaslioglu AA, Unlubilgin E, Aydogmus S, Keskin L, Dolen I (2012). A prospective randomized controlled trial of the transobturator tape and tissue fixation mini-sling in patients with stress urinary incontinence: 5-year results. J Urol.

[8] Petros PEP, Petros PEP (2010). The anatomy and dynamics of pelvic floor function and dysfunction. The female pelvic floor function, dysfunction and management according to the integral theory.

[9] Bump RC, Mattiasson A, Bo K (1996). The standardization of terminology of female pelvic organ prolapse and pelvic floor dysfunction. Am J Obstet Gynecol.

[10] Gin-Den Chen MD, Soo-Cheen Ng MD (2007). Updated definition of female pelvic organ prolapse. Incont Pelvic Floor Dysfunct.

[11] Altman D, Väyrynen T, Ellström Engh M, Axelsen S, Falconer C (2011). Anterior colporrhaphy versus transvaginal mesh for pelvic-organ prolapse. N Engl J Med.

[12] Nichols DH, Randall CL (1989) Massive eversion of the vagina. In: Nichols, Randall CL (eds) Vaginal surgery, 3rd edn. Williams Wilkins, Baltimore, pp 328–357

[13] Petros PE (2001). Vault prolapse I: dynamic supports of vagina. Int J Urogynecol Pelvic Floor Dysfunct.

[14] Petros PE, Ulmsten U, Papadimitriou J (1990). The autogenic neoligament procedure: a technique for planned formation of an artificial neo-ligament. Acta Obstet Gynecol Scand Suppl.

[15] Dietz HP, Shek C, De Leon J, Steensma AB (2008). Ballooning of the levator hiatus. Ultrasound Obstet Gynecol.

[16] Zacharin RF (1963). A suspensory mechanism of the female urethra. J Anat.

[17] Petros PE (2001). Vault prolapse II: restoration of dynamic vaginal supports by the infracoccygyeal sacropexy, an axial day-care vaginal procedure. Int J Urogynecol Pelvic Floor.

[18] Gordon AM, Huxley AF, Julian FJ (1966). The variation in isometric tension with sarcomere length in vertebrate muscle fibers. J Physiol.

[19] Milani R, Salvatore S, Soligo M, Pifarotti P, Meschia M, Cortese M (2005). Functional and anatomical outcome of anterior and posterior vaginal prolapse repair with prolene mesh. Br J Obstet Gynaecol.

[20] FDA (2008) Public health notification—serious complications associated with transvaginal placement of surgical mesh in repair of pelvic organ prolapse and stress urinary incontinence. US Food and Drug Administration, Silver Spring, MD 10.1016/j.eururo.2009.01.055 19645092

[21] Florey H, Florey H (1971). Inflammation. General pathology.

[22] Atherton MJ, Stanton SL (2005). The tension-free vaginal tape reviewed: an evidence-based review from inception to current status. Int J Obstet Gynaecol.

